# Prophylactic supplement with melatonin prevented the brain injury after cardiac arrest in rats

**DOI:** 10.1038/s41598-023-47424-x

**Published:** 2023-11-16

**Authors:** Yanan Hu, Xuyan Zhao, Ge Jiang, Mingxin Jin, Wei Jiang, Fei Han

**Affiliations:** 1 Department of Anesthesiology, The Third Medical Center of PLA General Hospital, Beijing, China; 2 Department of Anesthesiology, The Third Affiliated Hospital, Harbin Medical University, Harbin, Heilongjiang China

**Keywords:** Neuroscience, Cardiology, Diseases

## Abstract

Prophylactic pharmacotherapy for health care in patients with high risk of cardiac arrest (CA) is an elusive and less explored strategy. Melatonin has possibilities used as a daily nutraceutical to trigger the cellular adaptation. We sought to find the effects of long-term daily prophylactic supplement with melatonin on the victim of CA. Rats were divided into sham, CA, and melatonin + CA (Mel + CA) groups. The rats in the Mel + CA group received daily IP injection of melatonin 100 mg/kg for 14 days. CA was induced by 8 min asphyxia and followed by manual cardiopulmonary resuscitation. The endpoint was 24 h after resuscitation. Survival, neurological outcome, and hippocampal mitochondrial integrity, dynamics and function were assessed. Survival was significantly higher in the Mel + CA group than the CA group (81 vs. 42%, *P* = 0.04). Compared to the CA group, neurological damage in the CA1 region and the level of cytochrome c, cleaved caspase-3 and caspase-9 in the Mel + CA group were decreased (*P* < 0.05). Mitochondrial function and integrity were protected in the Mel + CA group compared to the CA group, according to the results of mitochondrial swelling, ΔΨm, ROS production, oxygen consumption rate, and respiratory control rate (*P* < 0.05). Melatonin increased SIRT3 and downregulated acetylated CypD. The mitochondrial dynamics and autophagy were improved in the Mel + CA group (*P* < 0.05). Long-term daily prophylactic supplement with melatonin buy the time from brain injury after CA.

## Introduction

Ischemia/reperfusion (I/R) injury after a cardiac arrest (CA) event is extremely damaging and critically time dependent^1,2^. Efforts to prevent, minimize, or reverse injury to vital organs are formidably challenging, with current resuscitation methods yield an average survival rate to hospital discharge with intact neurological function that approaches only 5% for out-of-hospital CA^3^. Cerebral recovery from long time CA was hampered by complex secondary derangements of multiple organ systems after reperfusion^4^. During prolonged transport or missed golden treatment window, the chance of good outcomes after restoration of spontaneous circulation (ROSC) was mainly depended on the enhanced endogenous defense mechanism and strong self-resistance ability conquering I/R stress. Long-term prophylactic pharmacotherapy for health care to buy the time for the victims from brain injury after CA is an elusive and less explored strategy. The development of prophylactic strategies with preservation of cerebral viability initiated and maintained during CA and cardiopulmonary resuscitation (CPR), and a delay of lethal cell injury targeting individuals at high-risk of suffering CA could lead to improvements in survival and neurological outcomes following such a devastating condition or in extreme environments.

Melatonin, classified as a dietary supplement for insomnia and jet lag, was an alternative, safer cardioprotective and cerebral protective supplement with few reported side-effects^5^. The importance of melatonin in nutrition was increasingly demonstrated for its properties of regulating physiological rhythm, scavenging free radical species, enhancing the immune system, showing anti-aging and anti-inflammatory effects, and performing anticancer activities, especially when taken during the day^6–8^. Evidence regarding the neuroprotective effects of applying melatonin intravenously or intraperitoneally as a therapeutic agent with prolonged survival, and improved structural and behavioral outcomes was obtained in experimental animals^9–11^. However, little is known about the influence of long-term daily prophylactic supplement with melatonin as a nutraceutical on preventing post-CA neurological deficits on the victims with high risk of CA. We hypothesized that long-term daily prophylactic supplement of melatonin was associated with increased survival, improved neurological function, and constituted an alternative preventive effect on mitochondrial dysfunction after resuscitation.

## Materials and methods

### Animals and experimental groups

The experimental protocol for the study was approved by the Institutional Animal Care and Use Committee of The Third Affiliated Hospital, Harbin Medical University and was performed in accordance with the NIH Guide for the Care and Use of Laboratory Animals. And the experiments reported were in compliance with the ARRIVE guidelines. Rats were euthanized by cervical dislocation after anesthetized with 2% sevofluorane.

**Figure 1:**
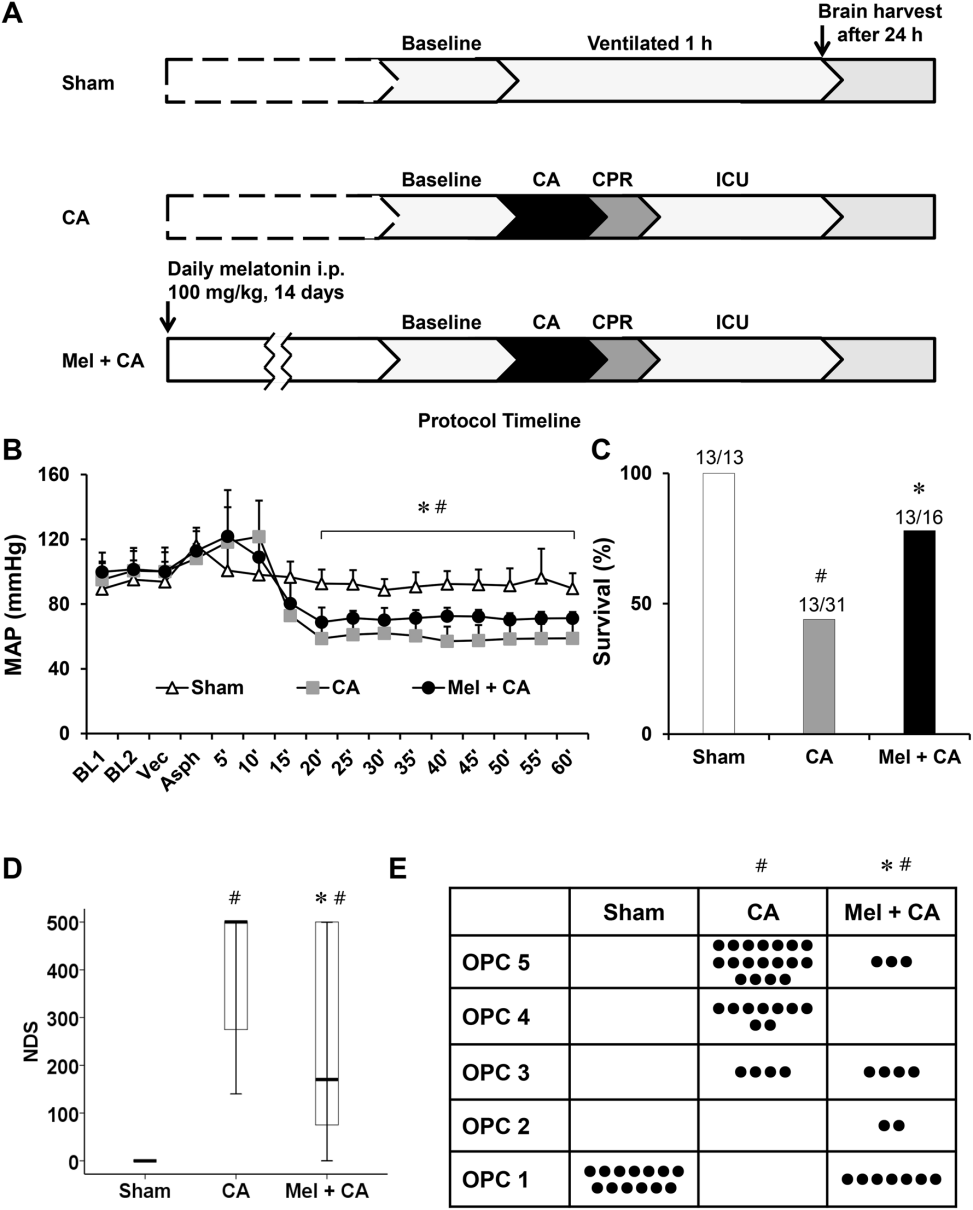
Physiological variables and neurological evaluation after asphyxial CA/CPR. (**A**), The experimental protocol. (**B**), MAP levels during CA, CPR and ROSC. One-way ANOVA followed by Bonferroni post hoc analysis was used. (**C**), Survival at 24 h after resuscitation. The chi-square test was used. (**D**), Overall performance categories (OPC, 1–5) at 24 h after resuscitation. The chi-square test was used. (**E**), Neurological deficit score (NDS, 0–500) at 24 h after resuscitation. Kruskal–Wallis test followed by Mann–Whitney U test were used. CA, cardiac arrest group; Mel + CA, melatonin + CA group (100 mg/kg i.p. for 14 days); MAP, Mean arterial pressure; CPR, cardiopulmonary resuscitation; ROSC, restoration of spontaneous circulation. * *P* < 0.05, vs. CA group; # *P* < 0.05, vs. sham group.

A total of 60 adult male Sprague–Dawley rats (weight 260–360 g) of 8–12 weeks were used for the study. They were housed in a rodent facility under 12 h light–dark cycle with unrestricted access to food and water. Rats were allocated into three groups: sham, CA, and melatonin plus CA (Mel + CA). Melatonin (N-acetyl-5-methoxytryptamine), dissolved in vehicle (2% dimethyl sulfoxide in sterile saline), was injected intraperitoneally to the rats in the Mel + CA group with a dosage of 100 mg/kg once daily at 10:00 a.m. for 14 days before the experiment (Fig. 1A). Rats in the CA group received the same volume of vehicle. The investigators performing CA and completing the evaluations were blinded to allocation assignment throughout the study period until final analysis.

### Asphyxia-induced CA and CPR

Following a 14-days administration of melatonin or vehicle, the experiment was performed at the same time of the day around 09:00–10:00 a.m. The experimental procedures were performed as previously described with minor modification^12^. Rats in the CA group and the Mel + CA group were anesthetized with 6% sevofluorane. Mechanical ventilation was initiated using a volume rodent ventilator (Model 683, Harvard Apparatus, South Natick, MA, USA) at the rate of 40/min, I:E = 1:1, FiO_2_ = 0.5. The tidal volume (8–12 ml/kg) was regulated by end-tidal CO_2_ maintained between 35 and 45 mmHg. Anesthesia was maintained with 1.5–2% sevofluorane. The left femoral artery and vein were cannulated for blood pressure monitoring and blood sampling. Rectal and tympanic probes were used to monitor the temperature. After the rats were equilibrated on the ventilator to get the baseline mean arterial pressure (MAP) and heart rate (HR), the experimental animals were given cisatracurium (1.5 mg/kg, intravenously) 5 min before asphyxia. CA was induced via asphyxia by turning off the ventilator and clamping the endotracheal tube. CA was confirmed by a MAP sharply decreased below 20 mmHg. The rectal temperature was maintained at 37.0 ± 0.5 °C during the entire asphyxia period by heating lamp. Following 8 min of asphyxia, chest compressions were performed at a rate of 200–300/min in combination with 0.01 mg/kg epinephrine, iv. Ventilation was resumed at the same time. Return of ROSC was confirmed by the return of HR rhythm and significant increase of MAP. If the rat does not establish ROSC after 5 min, efforts were stopped. The experimental animals maintained on mechanical ventilated after ROSC for 1 h. During this time, 1 μg epinephrine was used when MAP dropped below 50 mmHg. Rats received the same cannulation, anesthesia and infusions in the sham group. The endpoint was 24 h after resuscitation.

### Survival and neurological evaluation

Survival was compared at 24 h after resuscitation among groups. Revised neurological deficit scores (NDS; 0 = normal, 500 = brain death) and overall performance categories (OPC; 1 = normal, 2 = moderate disability, 3 = severe disability but conscious, 4 = coma, and 5 = death) were evaluated at 24 h post-resuscitation^12^.

### Tissue processing for histology

At 24 h after resuscitation, the survived rats were anesthetized and transcardially perfused via the left ventricle with 150 ml 0.9% iced saline followed by 100 mL of 4% paraformaldehyde. The brains were removed, post fixed in 4% paraformaldehyde overnight at 4 °C, and then transferred sequentially into 20%, and 30% sucrose solution overnight prior to dissection and sectioning. The brain tissue was embedded in Tissue-Tek® O.C.T. compound (Sakura Finetek Inc, Torrance, CA) and serially sectioned into 10 µm coronal sections on a cryostat (Leica, Germany). Sections were then collected into six-well plates containing PBS for histology.

### Nissl staining

Nissl staining was used to observe the morphological alterations in the hippocampus CA1 region. The sections were stained in 1% cresyl violet solution at 37 °C for 10 min and then were differentiated in 2.5% iced acetate ethanol solution for 20–30 min. The severity of neuronal damage was evaluated by counting the surviving neurons under a microscope and only neurons with distinct nucleus and nucleoli were considered as healthy living cells.

### Mitochondrial isolation and extraction

The brain was harvested at 24 h post-resuscitation. The hippocampal mitochondria were isolated from the brain. The highly purified mitochondria were used for western blot and analyses. For the mitochondrial function assay, the hippocampal mitochondria were extracted using a tissue mitochondria isolation kit (Beyotime Institute of Biotechnology, China). The homogenate of hippocampus was centrifuged at 1000 g for 5 min at 4 °C to remove nuclei and any unbroken cells. The supernatant was collected and centrifuged at 35,000 g for 10 min at 4 °C to obtain the mitochondrial fraction^13^.

### Transmission electron microscopy (TEM)

TEM analyses of mitochondria in the hippocampal neurons in the CA1 region were performed by an expert from the Electron Microscopy Laboratory of Harbin medical University. The hippocampal tissue acquired at 24 h after resuscitation were fixed in a solution of 4% paraformaldehyde and 0.5% glutaraldehyde/0.2 M cacodylate and then post-fixed with 1% osmium tetroxide and embedded in EmBed812^14^. Ultrastructures of the synapses in the hippocampal CA1 region were evaluated under TEM. The integrity of synaptic mitochondria, the synaptic vesicle density, and postsynaptic terminals were determined.

The standards for the evaluation of mitochondrial injury from grade 0 to 4 were as follows^15^: grade 0, normal mitochondria (mitochondria appeared highly dense with well-organized cristae); grade 1, early swelling as manifested by early clearing of matrix density and separation of cristae (a large amorphous matrix density and a linear density are present); grade 2, more marked swelling as manifested by further clearing of matrix density and separation of cristae; grade 3, more extensive mitochondrial swelling with disruption of cristae; grade 4, severe mitochondrial swelling with disruption of cristae and rupture of inner and outer mitochondrial membranes^15^. Normal (grade 0–1) and swollen mitochondria (grade 2–4) were counted with the Image J software (10 neuropil areas per group).

### Detection of ΔΨm

ΔΨm was monitored using the JC-1 Mitochondrial Membrane Potential Detection Kit (Beyotime Institute of Biotechnology, China). ΔΨm was determined using the ratio of JC-1 aggregates (red) to JC-1 monomers (green). Mitochondrial depolarization was expressed as the decrease in the intensity ratio of red/green fluorescence^16^.

### Detection of ROS

Mitochondrial ROS was measured with a ROS assay kit (GENMED Bioengineering Institute, Shanghai, China). The chromogenic reaction mixture was added to equal amounts of mitochondria (50 μg/50 μL) in each sample at 37 °C for 15 min. All steps were performed in the dark. Production of ROS was observed at an excitation and emission of 490 nm and 530 nm with an epifluorescence microscope^16^.

### Mitochondrial oxygen consumption

Oxygen consumption by mitochondria (1 mg/mL) was measured with a Clark-type oxygen electrode at 25 °C (Hansatech Oxygraph, Hansatech, Norfolk, UK). The baseline oxygen consumption rate (OCR) was measured at 2 min following the addition of mitochondria until the recorded curve stabilized. State 4 respiration was initiated by 20 μL disodium succinate (4 mM). Then, 20 μL adenosine diphosphate (ADP, 50 mM) was added to the incubation medium to initiate state 3 respiration. The respiratory control rate (RCR) was calculated from the rate of state 3 to state 4 respiration^17^.

### Immunofluorescence staining

Immunofluorescence staining was performed as described previously. In brief, after being blocked with goat serum for 1 h at 37 °C, the sections were incubated with rabbit anti-SIRT3 (1:50, Santa Cruz Biotechnology) or rabbit anti-cytochrome c (1:50, Santa Cruz Biotechnology) overnight at 4 °C and then with secondary antibody (CY3-conjugated rabbit anti-mouse lgG, Beyotime) for 1 h at room temperature. Nuclei was labeled with 4,6-diamidino-2-phenylindole (DAPI; 1:1000, Beyotime) for 1 min. Images were captured with a confocal fluorescence microscope (Olympus, Tokyo, Japan).

### Western blot analysis

The frozen hippocampus samples in each group were homogenized and protein was quantified by BCA Protein Assay kit (Beyotime Biotechnology). Western blot was performed as previously described^18^. Images of blots were captured with an Image Quant ECL Imager (GE Healthcare, Chicago, IL), and the bands were quantified with Image J software. The following primary antibodies were used: cleaved caspase-3 (1:500, Cell Signaling Technology), caspase-9 (1:500, Cell Signaling Technology), Cyclophilin D (CypD; 1:500, Abcam), mitofusin 2 (Mfn2; 1:1000, Abcam), mitochondrial dynamin-related protein 1 (Drp1; 1:500, Abcam), PTEN-induced putative kinase 1 (PINK1; 1:500, Abcam), Parkin (1:500, Abcam), LC3 (1:400, Abcam), and β-actin (1:2000, Abcam). Acetylation of CypD was determined by immunoprecipitation followed by western blot analysis. Acetylated CypD was quantified by normalization with IgG.

### Statistical analysis

Sample size for CPR outcome measurements was at least twelve rats per group with an alpha error of 0.05 and a power of > 80%. The physiological parameters, the mitochondrial integrity, the mitochondrial swelling, ΔΨm, ROS, and immunofluorescence were analyzed by a one-way ANOVA followed by Bonferroni post hoc analysis or a Student’s t-test. The MAP was analyzed by repeated measures analysis of ANOVA. The chi-square test was used to test the rates of ROSC, the survival, the differences in proportions of OPC values between groups (favorable vs. unfavorable outcome, OPC 1–2 vs. OPC 3–5). Kruskal–Wallis test followed by Mann–Whitney U test were used to compare NDS among groups. One-way ANOVA followed by LSD post hoc tests was performed to identify differences between groups in the neuronal cell loss, mitochondrial oxygen consumption parameters and to evaluate the western blot quantification analysis. A *P* < 0.05 was considered a significant difference. Statistical tests were performed with SPSS 19.0 software (SPSS Inc., USA).

### Ethics approval and consent to participate

The experimental protocol for the study was approved by the Institutional Animal Care and Use Committee of The Third Affiliated Hospital, Harbin Medical University and was performed in accordance with the NIH Guide for the Care and Use of Laboratory Animals.

## Results

There were no differences in body weight, HR, and body temperature among groups at baseline (Table 1). The duration from asphyxia to CA, the duration of CPR, and dosages of epinephrine were no differences among groups. MAP after resuscitation was lower in the CA group than the sham group (*P* < 0.05, Fig. 1B). MAP after resuscitation was higher in the Mel + CA group than the CA group (*P* < 0.05). There are 13 rats in each group survived successfully at 24 h after resuscitation. The resuscitated rats were used for immunofluorescence staining (n = 3), Nissl staining (n = 3), TEM analysis (n = 3), mitochondria measurement (n = 6), and western blot analysis (n = 3).

**Table 1:** Baseline characteristics, duration of CA and CPR, and dosage of epinephrine.

	Sham	CA	Mel + CA	*P* value
Body weight (g)	317.4 ± 24.3	330.9 ± 24.3	323.7 ± 26.2	0.207
Heart rate (bpm)	331.9 ± 7.5	327.9 ± 6.7	332.7 ± 9.1	0.246
Body temperature (°C)	36.8 ± 0.5	36.5 ± 1.2	36.4 ± 0.4	0.801
Duration of CA (s)	–	191.3 ± 26.1	186.2 ± 22.3	0.559
Duration of CPR (s)	–	36.2 ± 11.2	45.1 ± 30.6	0.328
Dosage of epinephrine (mg)	–	3.31 ± 0.2	3.23 ± 0.2	0.247

Data are shown as mean ± SD. One-way ANOVA followed by Bonferroni post hoc analysis or a Student’s t-test was used. CA: cardiac arrest, CPR: cardiopulmonary resuscitation, ROSC, restoration of spontaneous circulation.

### Melatonin improved survival and neurological outcome

The ROSC rate did not differ among groups (CA = 70.4%, and Mel + CA = 64.0%, *P* = 0.60). All rats in the sham group survived. The 24 h survival was significantly higher in the Mel + CA group (81%, 13 of 16) than the CA group (41%, 13 of 31) (*P* = 0.01, Fig. 1C). According to the lower survival for post resuscitation 24 h in the CA group (41%) compared to the other groups in our study, the sample size of each group was various to ensure enough samples acquired for mitochondrial function measurements.

Neurological outcome and overall recovery were significantly better in the Mel + CA group than the CA group (NDS, *P* < 0.001, OPC, *P* < 0.001, Fig. 1D and E). There were no differences among other groups.

### Melatonin reduced neurological damage

**Figure 2:**
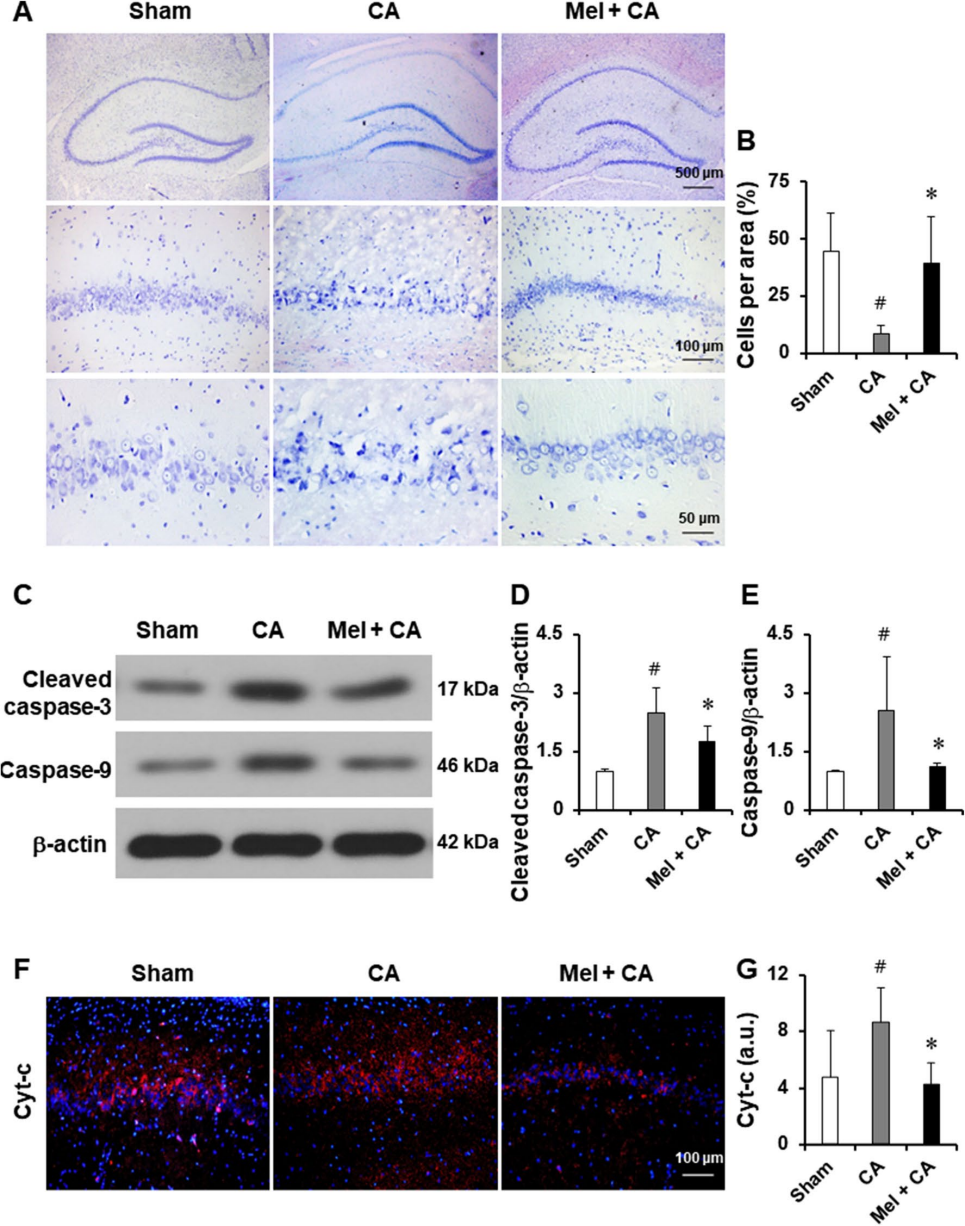
Melatonin reduced neurological damage. A-B, Representative Nissl staining at 24 h after resuscitation and quantitative analysis of the number of positive cells in the CA1 region of hippocampus (n = 3 per group). C-D, Immunofluorescent staining and quantitative analysis of Cyt-c in the CA1 region of hippocampus (n = 3 per group). E–G, Representative western blot and quantitative analysis of cleaved caspase-3 and caspase-9. Data are mean ± SD (n = 3 per group) obtained from different tissue preparations. One-way ANOVA followed by Bonferroni post hoc analysis or LSD post hoc tests were used. Cyt-c, Cytochrome c. * *P* < 0.05, vs. CA group; # *P* < 0.05, vs. sham group.

The neurological damage was evaluated by Nissl staining (Fig. 2A). Neuronal cell loss and neuronal pyknosis in CA1 region of hippocampus was significantly increased in the CA group than the sham group (*P* = 0.01, Fig. 2B). Neuronal cell loss and neuronal pyknosis was no difference between the Mel + CA group and the sham group. Neuronal cell loss and neuronal pyknosis was significantly reduced in the Mel + CA group than the CA group (*P* = 0.04).

Cleaved caspase-3 and caspase-9 expressed more in the CA group than the sham group (*P* = 0.001, *P* = 0.022, Fig. 2C). Cleaved caspase-3 and caspase-9 expression was no difference between the Mel + CA group and the sham group. Cleaved caspase-3 and caspase-9 were expressed less in the Mel + CA group than the CA group (*P* = 0.043, *P* = 0.031).

### Melatonin reduced the cytochrome c (Cyt-c) release

Immunoreactivity of Cyt-c for the hippocampus was significantly increased in the CA group than the sham group (*P* = 0.013, Fig. 2F). Immunoreactivity of Cyt-c was no difference between the Mel + CA group and the sham group. Immunoreactivity of Cyt-c was lower in the Mel + CA group than the CA group (*P* = 0.002).

### Melatonin maintained mitochondrial ultrastructure and integrity

**Figure 3:**
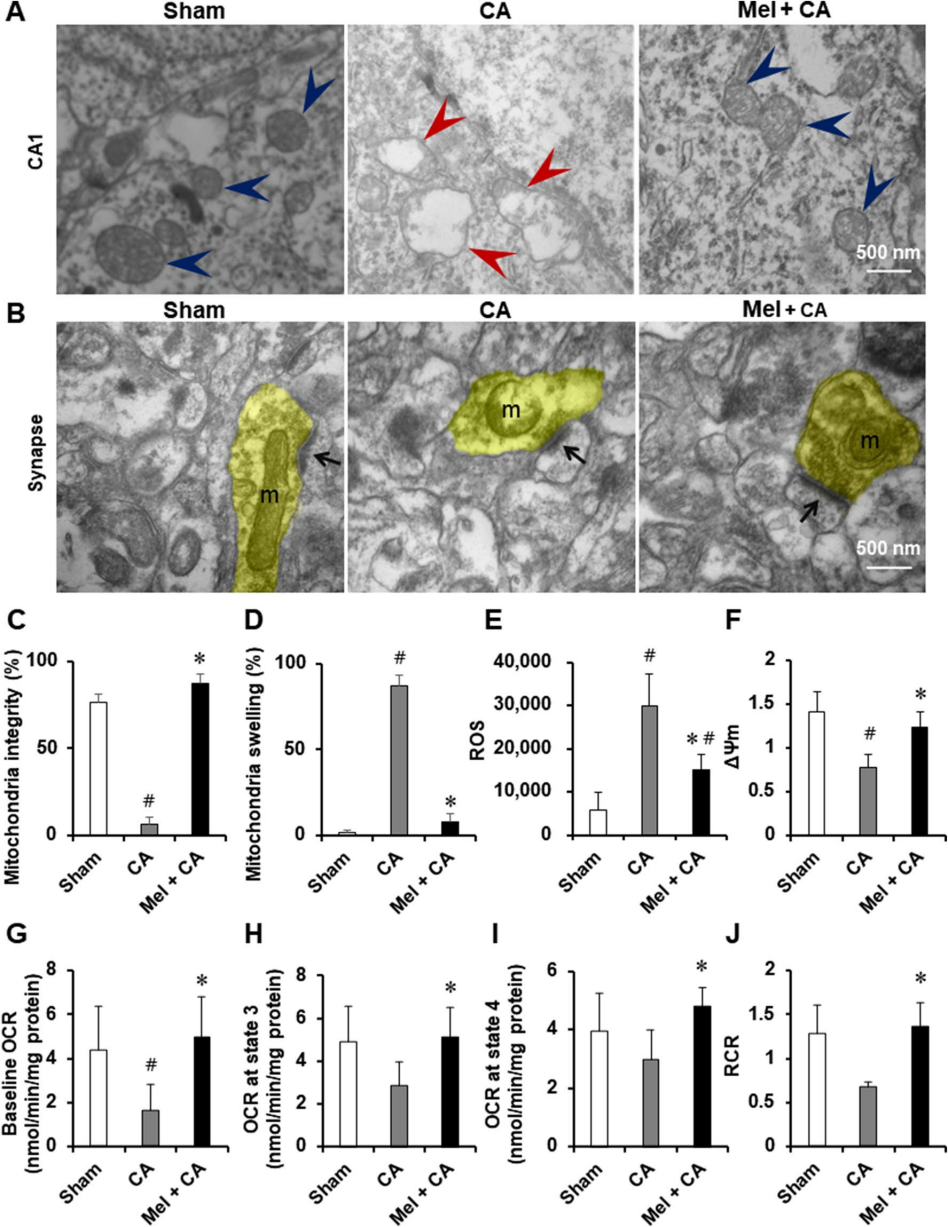
Melatonin maintained mitochondrial function and ultrastructure intersity. (**A**, **B**), Transmission electron microscopy images showing the ultrastructure changes of mitochondria in the CA1 region and synapses of hippocampus. Blue arrowheads, mitochondria exhibited normal membrane integrity and no injury; red arrowheads, mitochondria exhibited swelling, decreased matrix density, and cristae disintegration; arrows, postsynaptic density; yellow shading, presynaptic terminal. (**C**), Mitochondria integrity. Each data point is the mean of n = 10 neurons per brain; 3 brains per group. (**D**), Mitochondria swelling. E, ΔΨm. F, ROS. G, Baseline OCR. H, OCR at state 3. I, OCR at state 4. J, RCR. One-way ANOVA followed by Bonferroni post hoc analysis or LSD post hoc tests were used. OCR, oxygen consumption rate; RCR, respiratory control rate (state 3/state 4). * *P* < 0.05, vs. CA group; # *P* < 0.05, vs. sham group.

Ultrastructural changes of the mitochondria in the hippocampus CA1 region were shown in Fig. 3A. Mitochondria in the sham group exhibited normal membrane integrity. Mitochondria in the CA group were severely damaged and exhibited swelling, decreased matrix density and collapsed cristae. Mitochondria in the Mel + CA group showed no signs of swelling and injury. The number of normal mitochondria in the CA group was significantly decreased than the sham group (*P* < 0.001). The number of normal mitochondria was no difference between the Mel + CA group and the sham group. The number of normal mitochondria in the Mel + CA group was significantly increased than the CA group (*P* < 0.001).

The morphological alterations of synapse in the hippocampus CA1 region were observed using TEM (Fig. 3B). Inflated mitochondria with broken membrane integrity, reduced synaptic vesicle density, and decreased postsynaptic terminals were showed in the CA group. No obvious signs of degeneration (synaptic vesicle, inflated mitochondria, diffuse vacuolization) were detected in the Mel + CA group.

### Melatonin maintained mitochondrial function

Mitochondrial ROS were significantly increased 24 h after resuscitation in the CA group than the sham group (*P* < 0.001, Fig. 3E). Mitochondrial ROS was less produced in the Mel + CA group than the sham group (*P* < 0.001). Mitochondrial ROS was less produced in the Mel + CA group than the CA group (*P* = 0.018).

ΔΨm in the CA group was significantly decreased than the sham group (*P* < 0.001, Fig. 3F). ΔΨm was no difference between the Mel + CA group and the sham group. ΔΨm was higher in the Mel + CA group than the CA group (*P* = 0.004).

The OCR at baseline and state 3 in the CA group were significantly lower than the sham group (*P* = 0.024, *P* = 0.048, Fig. 3G–I). The OCR at baseline, state 3, state 4 and RCR was no difference between the Mel + CA group and the sham group. The OCR at baseline, state 3, state 4 and RCR in the Mel + CA group were significantly higher than the CA group (*P* = 0.005,* P* = 0.031,* P* = 0.032, and *P* = 0.023). The mitochondrial OCR measuring mitochondrial oxidative phosphorylation activity, indicated the quantitative relationship between mitochondrial respiration and cellular function^19^. The improved OCR showed that supplementation with melatonin ameliorated the decline in mitochondrial functional capacity exposed to I/R injury by enhancing the ATP content, and ATP production rate.

### Melatonin inhibited acetylated CypD and downregulated SIRT3 level

**Figure 4:**
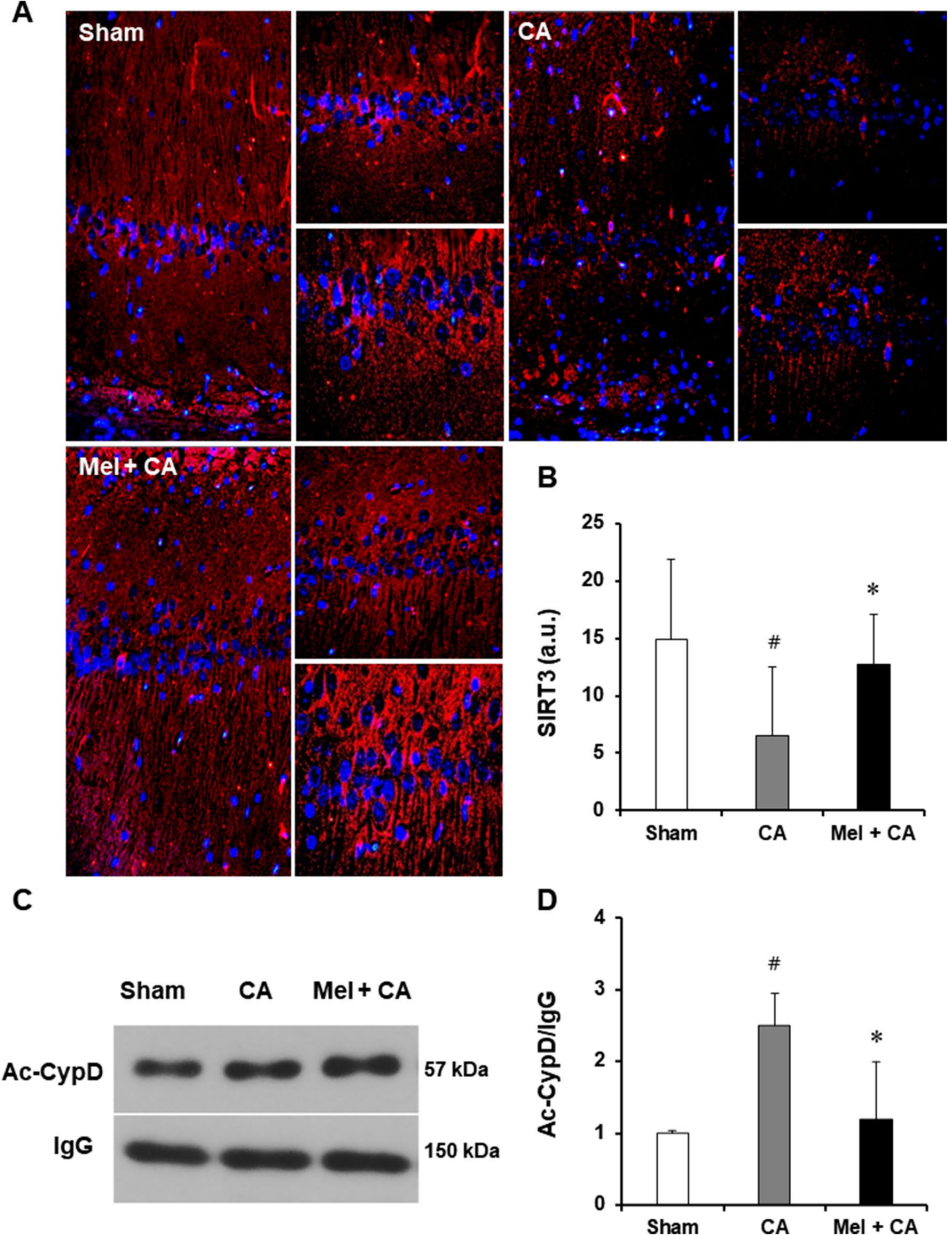
Melatonin inhibit acetylated CypD via downregulating SIRT3 expression. (**A**, **B**), Immunofluorescent staining of SIRT3 (red) in the hippocampus. Nuclei were counterstained with DAPI (blue). (**C**, **D**), Representative western blot and quantitative analysis of Ac-CypD. Data are mean ± SD (n = 3 per group) obtained from different tissue preparations. One-way ANOVA followed by Bonferroni post hoc analysis or LSD post hoc tests were used. *P < 0.05, vs. CA group; # *P* < 0.05, vs. sham group.

Immunofluorescence assays showed the level of SIRT3 in CA1 region of the hippocampus (Fig. 4A). The level of SIRT3 was significantly decreased in the CA group than the sham group (*P* = 0.02). The level of SIRT3 was no difference between the Mel + CA group and the sham group. The level of SIRT3 was significantly increased in the Mel + CA group than the CA group (*P* = 0.03).

The protein level of acetylated CypD in the CA group was higher than the sham group (*P* = 0.01, Fig. 4B). The acetylated CypD expression was no difference between the Mel + CA group and the sham group. The protein level of acetylated CypD in the Mel + CA group was significantly lower than the CA group (*P* = 0.03).

### Melatonin modulated mitochondrial dynamics signaling and mitophagy proteins

**Figure 5:**
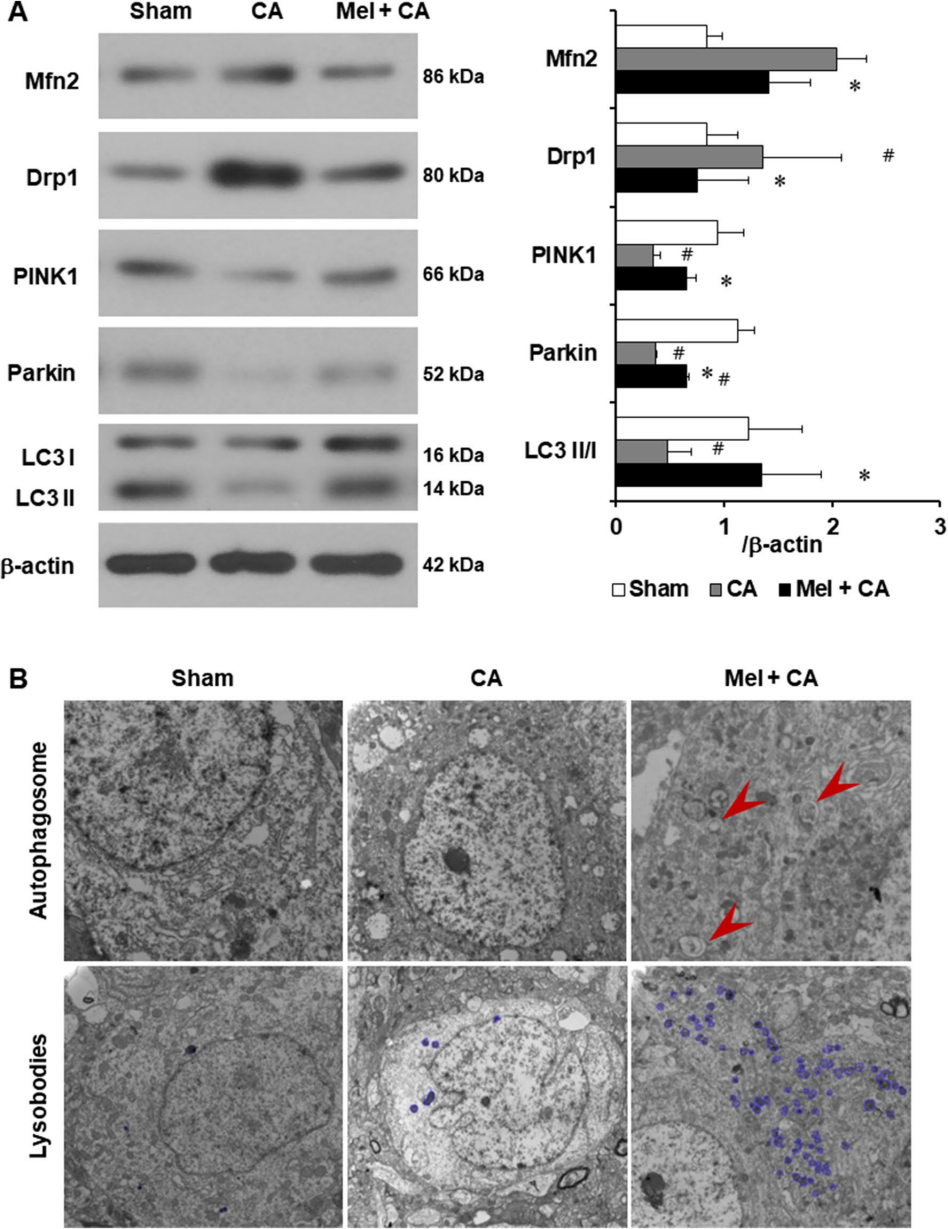
Melatonin modulated mitochondrial dynamics signaling and mitophagy proteins. (**A**), Representative western blot and quantitative analysis of Mfn2, Drp1, PINK1, Parkin, and LC3 II/I (n = 3 per group). One-way ANOVA followed by LSD post hoc tests was used. (**B**), Transmission electron microscopy images of the autophagosome formation (red arrowheads) and lysobodies (blue) in the hippocampal CA1 neurons. Autolysosomes were not observable in the sham and the CA groups. Autolysosomes were visible in the Mel + CA group. Compared to the sham and the CA groups, increased lysobodies were observed in the Mel + CA group. *P < 0.05, vs. CA group; # *P* < 0.05, vs. sham group.

The fusion-and fission-related proteins of Mfn2 and Drp1 were higher in the CA group than the sham group (*P* = 0.062, *P* = 0.003, Fig. 5A). The protein levels of Mfn2 and Drp1 were significantly lower in the Mel + CA group than the CA group (*P* = 0.039, *P* = 0.013). The mitophagy-related proteins of PINK1 and Parkin were significantly lower in the CA group than the sham group (*P* = 0.003, *P* < 0.001). The protein level of Parkin was significantly lower in the Mel + CA group than the sham group (*P* < 0.001). The protein levels of PINK1 and Parkin were significantly higher in the Mel + CA group than the CA group (*P* = 0.048, *P* = 0.011). The autophagy-related protein of LC3 was quantified by LC3 II (an active form) to LC3 I (a cytosolic form). The ratio of LC3 II/I was significantly lower in the CA group than the sham group (*P* = 0.048). The ratio of LC3 II/I was significantly higher in the Mel + CA group than the CA group (*P* = 0.040). There were no differences among other groups.

TEM images showed the ultrastructure findings of autophagosomes and lysosomes in the hippocampus of three groups (Fig. 5B). In the CA group, no visible autophagosomes and less lysosomes were observed. In the Mel + CA group, round autophagosomes were located next to nuclei and there were abundant lysosomes. Some residual mitochondria coated by autophagosomes were detectable, and autophagy was evident.

## Discussion

To our knowledge, this is the first study systematically exploring a long-term prophylactic strategy to conquer the extreme physiological challenges and detrimental effects after CA. Our data clearly demonstrated that administration with melatonin for 14 days in rats resulted in significant improvement of survival, markedly alleviated neurological damage, and significant preservation of mitochondrial intensity and function after CA. The melatonin induced a potential cellular adaptive response to ischemia and promoted pro-survival pathway by increasing capacity for mitochondrial biogenesis.

The timing of melatonin administration in the present study provided a new insight on its therapeutic window for improving post-resuscitation survival. Unexpected occurrence and lack of the prediction of CA onset make its prevention difficult. Most neuroprotective drugs were commonly active on the immediate or remote clinical effects after CA, ineffectively in improving survival or neurologic outcome^20^. These drugs were limited by the medical given time and delays common in all phases of public CA process, including the initiation of bystander CPR, notification of Emergency Medical Service (EMS) providers, and transportation to hospital^21^, during which the global ischemia sensitizes systems regulating stress responses persisted over time, resulting in allostatic loads with irreversible brain injury and maladaptive physiological responses^22^. The strategy in our study that daily melatonin supplement applied at a time period of before CA confirmed its possibility to rescue the brain from time, but it’s quite challenging for no other intervention applied during CA. In the similar condition of hibernation, melatonin helped the hibernator adapted to the physiological extreme changes in body temperature and tolerated of the subsequently cerebral IR injury^23^. Especially recent study suggested that under appropriate conditions, the protective mechanism were partially restored after a prolonged post-CA interval even in the absence of protection measures^24^. At the stage of 24 h post-resuscitation, the functional proteins and process were comprehensively downregulated with time^25^. We observed that melatonin restored approximately 80% of the hippocampal CA1 region neurons, suggesting the strategy was useful to prevent the lethal injury of CA.

The positive effects of melatonin were associated with cellular-level changes focusing on the opening of the mitochondrial permeability transition pore (mPTP) with preserved ΔΨ, and less matrix swelling, and the stimulation of a dynamic virtuous cycle of mitochondrial biogenesis, mitophagy and autophagy by coactivating these proteins import. It was remained controversial of the dual role of autophagy in cell survival or death which depended on the severity of cellular damage^26^. Roohbakhsh, A et al. believed that melatonin may affect mechanisms that stimulate autophagy, rather than affecting the process itself^27^. The robust ineversible mPTP opening was the downstream executive event of necroptosis which was particularly critical to neuronal survival^28^. We determined the CypD acetylation, which was known to be a direct activator of mPTP^29,30^, to confirm the melatonin’s protection effect. But the connection between melatonin and SIRT3/CypD in cerebral I/R injury seemed not-so unexpected. As SIRT3 was involved in diversified biological processes, the involvement of other pathways in the future study could enrich the more comprehensive understanding of the benefits of melatonin administration.

Another factor to consider is the optimal duration of melatonin supplement that may be effective. As a health care products or even a partner with aspirin, the preventive effect of dietary melatonin intake or contained in Mediterranean diets on cardiovascular and neurodegenerative diseases was known appeared days and weeks^31^. Deep understanding the multiple prophylactic strategy, mitochondria emerged as the final common target for melatonin. Report about subcellular distribution described that there actually exert a movement to mitochondria when treatment with melatonin^32^. Under the burst signal reactive event, our findings describe for the first time that a higher dosage of daily melatonin for 14 days protected cells from extremely high oxidative stress triggered by CA, and reduced the stimuli sources of mitochondrial dysfunction including the ROS production and ATP depletion, with preserved mitochondrial respiratory capacity. It was complex to explain whether the higher concentration of melatonin or a preserved melatonin rhythm worked. Literatures show that melatonin in other body fluids and cells are not necessarily in equilibrium with those in the blood^33^. While Kerry Rennie et al. suggested that persistently high levels of melatonin in the brain exerted long-term neuroprotection against ischemia^34^. Another view is that the preserved higher melatonin rhythm reduced oxidative damage to a greater degree than the commonly accepted lower amplitude rhythm^35^. The future study can expand around this point.

The results of our preliminary study indicated that prophylactic low-dosage (10 mg/kg, 20 mg/kg) melatonin had no significant effect on neuronal injury after CA. The dosage of melatonin used in previous study in ischemia perfusion rats was ranged from 2.5 to 300 mg/kg, and the dosage of melatonin 100 mg/kg used in CA model demonstrated effectiveness^17,36^. Therefore, a dosage of 100 mg/kg was selected for this study. Also the safety was showed in the study that chronic rectal melatonin with up to 300 mg/day was well tolerated during an observation period of up to 2 years^37^.

There are several important limitations in the present study. First, although the results were promising, the endpoint was relatively short (1 day after resuscitation). Based on the clinical data that the I/R injury persisted for at least 1 week after established reperfusion, a longer observation period was need to further explore the neurological benefits from prophylactic melatonin supplement. Second, this study was not based on a random grouping method. Although the sample size was enough, the sample for some histological and biochemical determinations was low and bias was inevitable. Third, the study only determined the expression of mitochondrial dynamics proteins, but how melatonin directly affect mitochondrial dynamics was unclear. It could be explored in a cellular model of IR in future study. Fourth, there were no data on the pharmacokinetics and toxicities of melatonin after injected intraperitoneally in rat. A melatonin only group could better elucidate its safety including the influence on behavioral status and alertness in such a high dosage (Supplementary Information).

## Conclusions

Our findings, for the first time, suggest that long-term daily prophylactic melatonin supplement enhanced the innate capacity to buy the time for the victims from brain injury after CA. The prophylactic strategy with melatonin is useful in improving survival, preventing functional neurological damage after CA, and protecting against mitochondrial dysfunctions.

### Supplementary Information

Supplementary Information.

## Data Availability

The data that support the findings of this study are available from the corresponding author upon reasonable request.

## Abbreviations

I/R: Ischemia/reperfusion
CA: Cardiac arrest
ROSC: Restoration of spontaneous circulation
CPR: Cardiopulmonary resuscitation
MAP: Mean arterial pressure
HR: Heart rate
NDS: Neurological deficit scores
OPC: Overall performance categories
DAPI: 4,6-diamidino-2-phenylindole
TEM: Transmission electron microscopy
OCR: Oxygen consumption rate
ADP: Adenosine diphosphate
RCR: Respiratory control rate
CypD: Cyclophilin D
Mfn2: Mitofusin 2
Drp1: Dynamin related protein 1
PINK1: PTEN-induced putative kinase 1
Cyt-c: Cytochrome c
mPTP: Mitochondrial permeability transition pore
