# Supplementary material for: Prophylactic supplement with melatonin prevented the brain injury after cardiac arrest in rats
Source: Sci Rep. 2023 Nov 16;13:20100. doi: 10.1038/s41598-023-47424-x (PMC10654502; doi:10.1038/s41598-023-47424-x)
Supplement: Supplementary file 1 — Supplementary Information. [file 41598_2023_47424_MOESM1_ESM.pptx]

## Slide 1
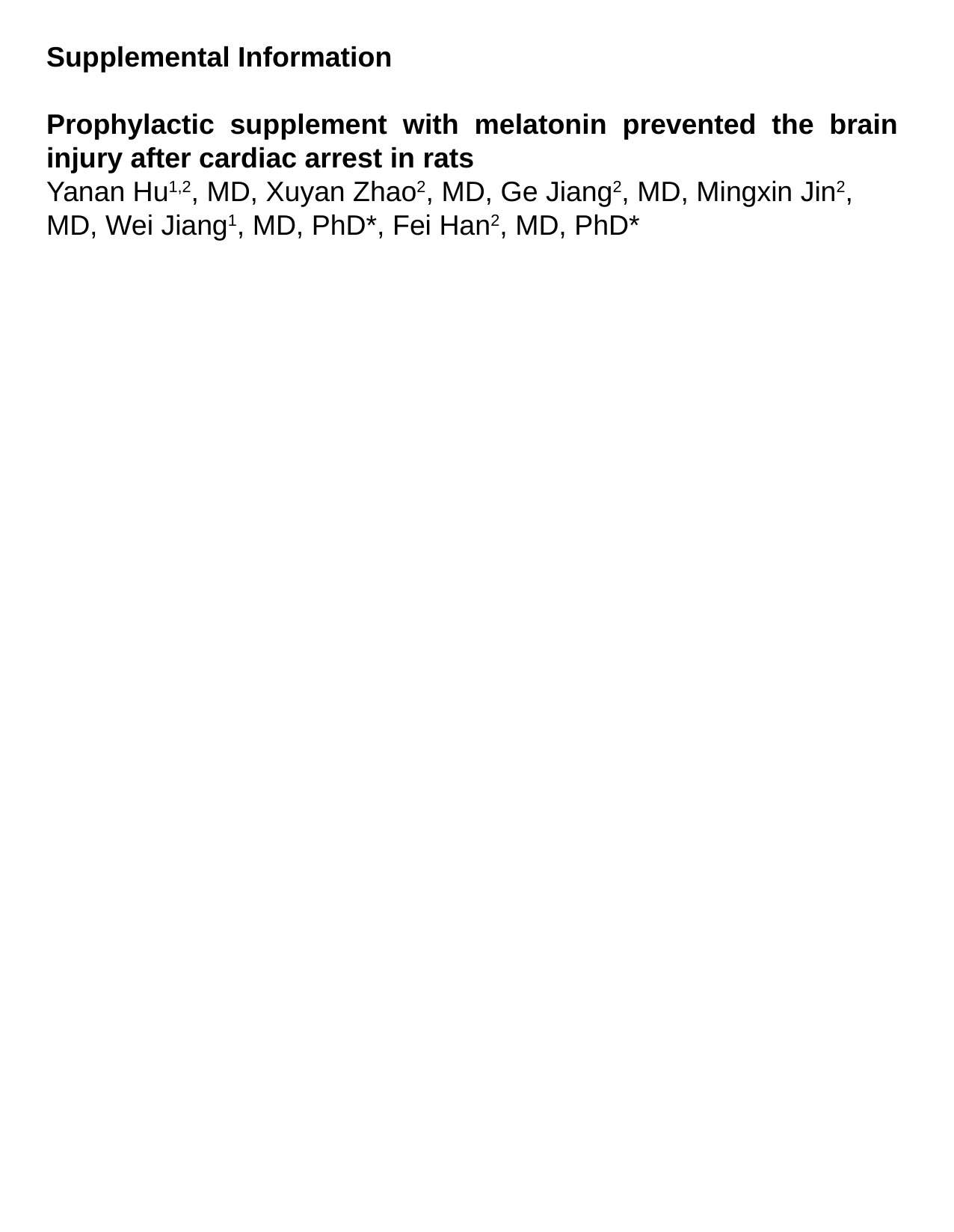

Supplemental Information
Prophylactic supplement with melatonin prevented the brain injury after cardiac arrest in rats
Yanan Hu1,2, MD, Xuyan Zhao2, MD, Ge Jiang2, MD, Mingxin Jin2, MD, Wei Jiang1, MD, PhD*, Fei Han2, MD, PhD*

## Slide 2
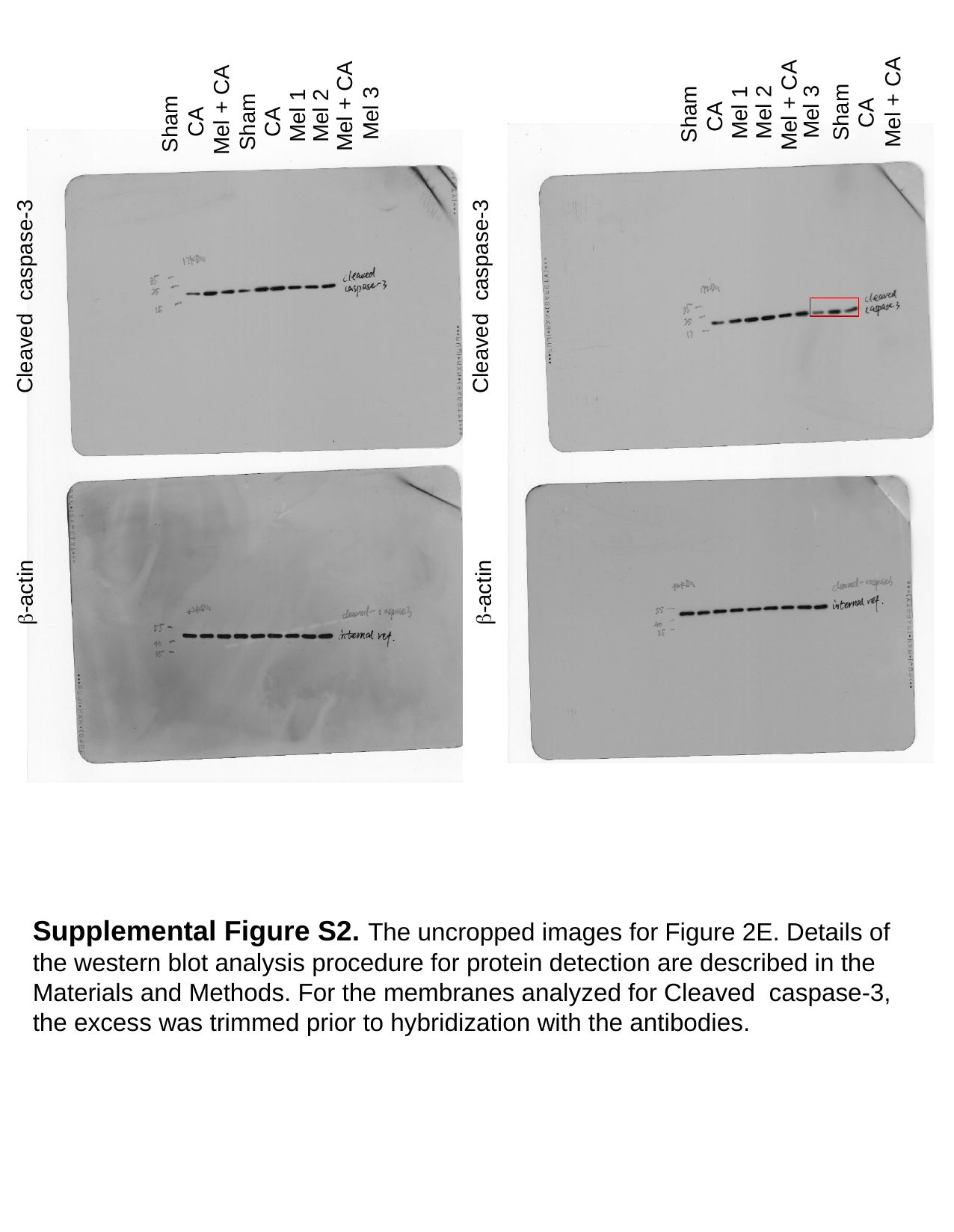

Mel + CA
Mel + CA
Mel 3
Mel 2
CA
Sham
Mel 1
Sham
CA
Mel + CA
Mel + CA
Mel 3
Mel 1
Mel 2
Sham
CA
CA
Sham
Cleaved caspase-3
Cleaved caspase-3
β-actin
β-actin
Supplemental Figure S2. The uncropped images for Figure 2E. Details of the western blot analysis procedure for protein detection are described in the Materials and Methods. For the membranes analyzed for Cleaved caspase-3, the excess was trimmed prior to hybridization with the antibodies.

## Slide 3
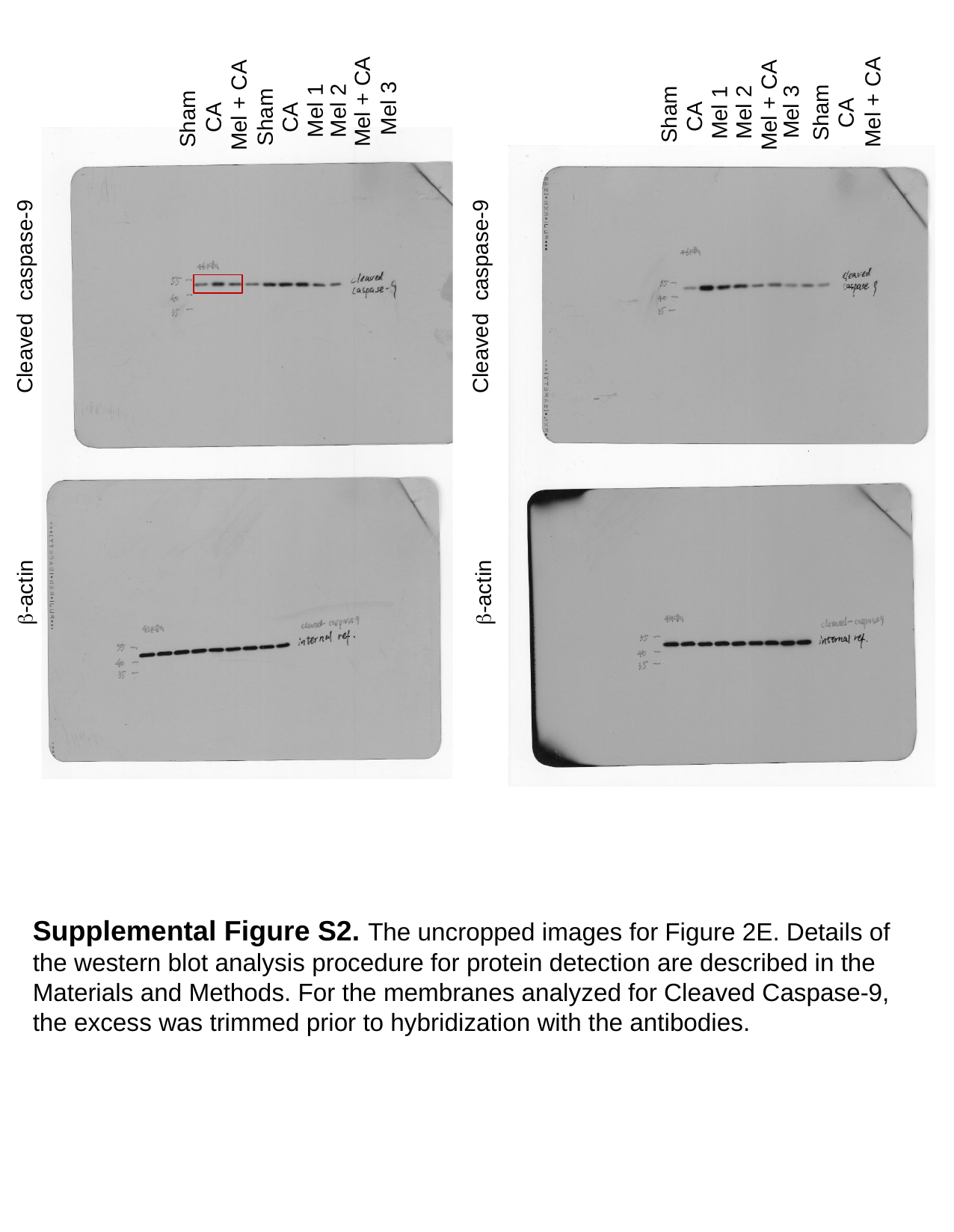

Mel + CA
Mel + CA
Mel 3
Mel 1
Mel 2
Sham
CA
CA
Sham
Mel + CA
Mel + CA
Mel 3
Mel 2
CA
Sham
Mel 1
Sham
CA
Cleaved caspase-9
Cleaved caspase-9
β-actin
β-actin
Supplemental Figure S2. The uncropped images for Figure 2E. Details of the western blot analysis procedure for protein detection are described in the Materials and Methods. For the membranes analyzed for Cleaved Caspase-9, the excess was trimmed prior to hybridization with the antibodies.

## Slide 4
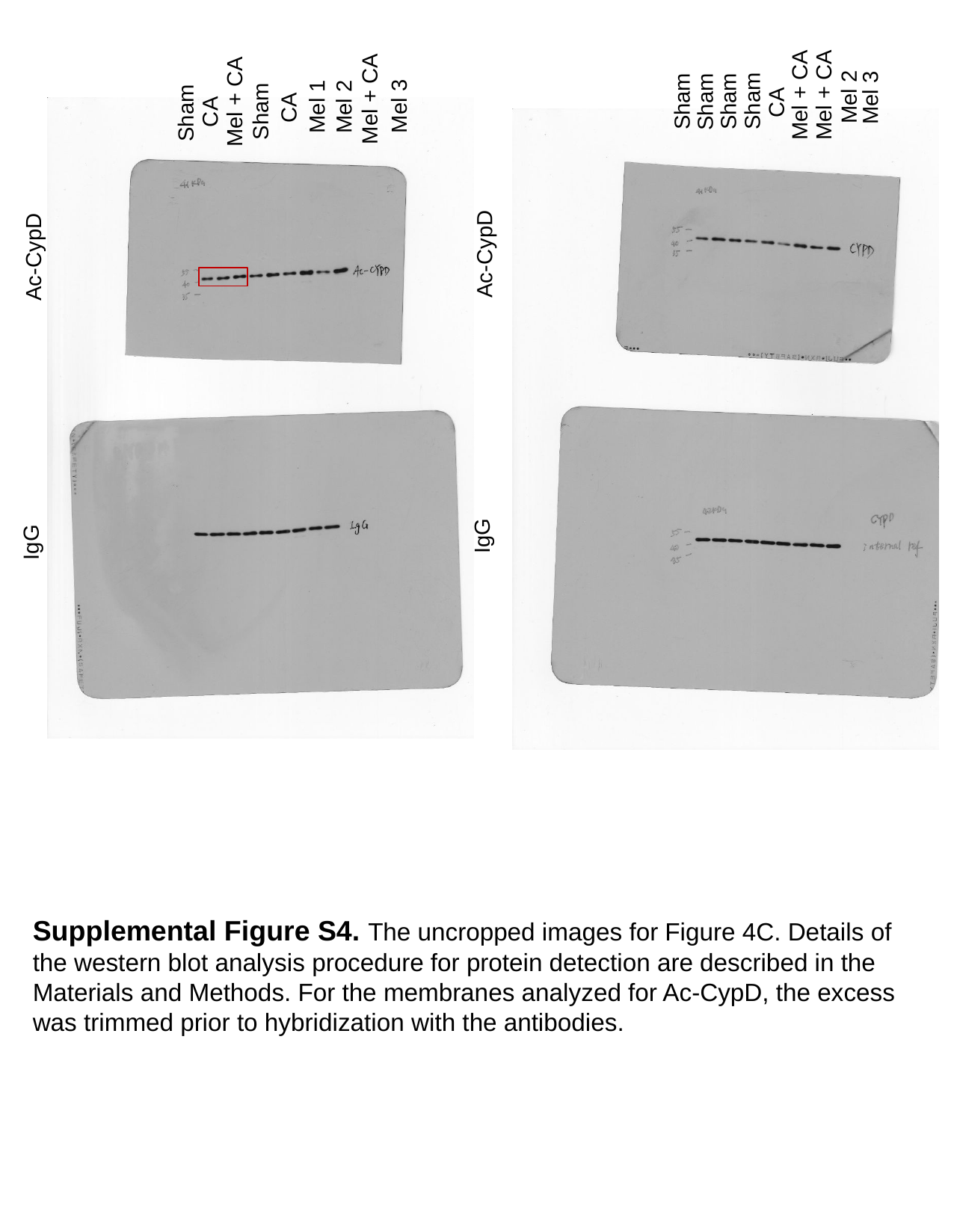

Mel + CA
Sham
Sham
Sham
Sham
CA
Mel + CA
Mel 2
Mel 3
Mel + CA
Mel + CA
Mel 3
Mel 1
Mel 2
CA
Sham
CA
Sham
Ac-CypD
Ac-CypD
IgG
IgG
Supplemental Figure S4. The uncropped images for Figure 4C. Details of the western blot analysis procedure for protein detection are described in the Materials and Methods. For the membranes analyzed for Ac-CypD, the excess was trimmed prior to hybridization with the antibodies.

## Slide 5
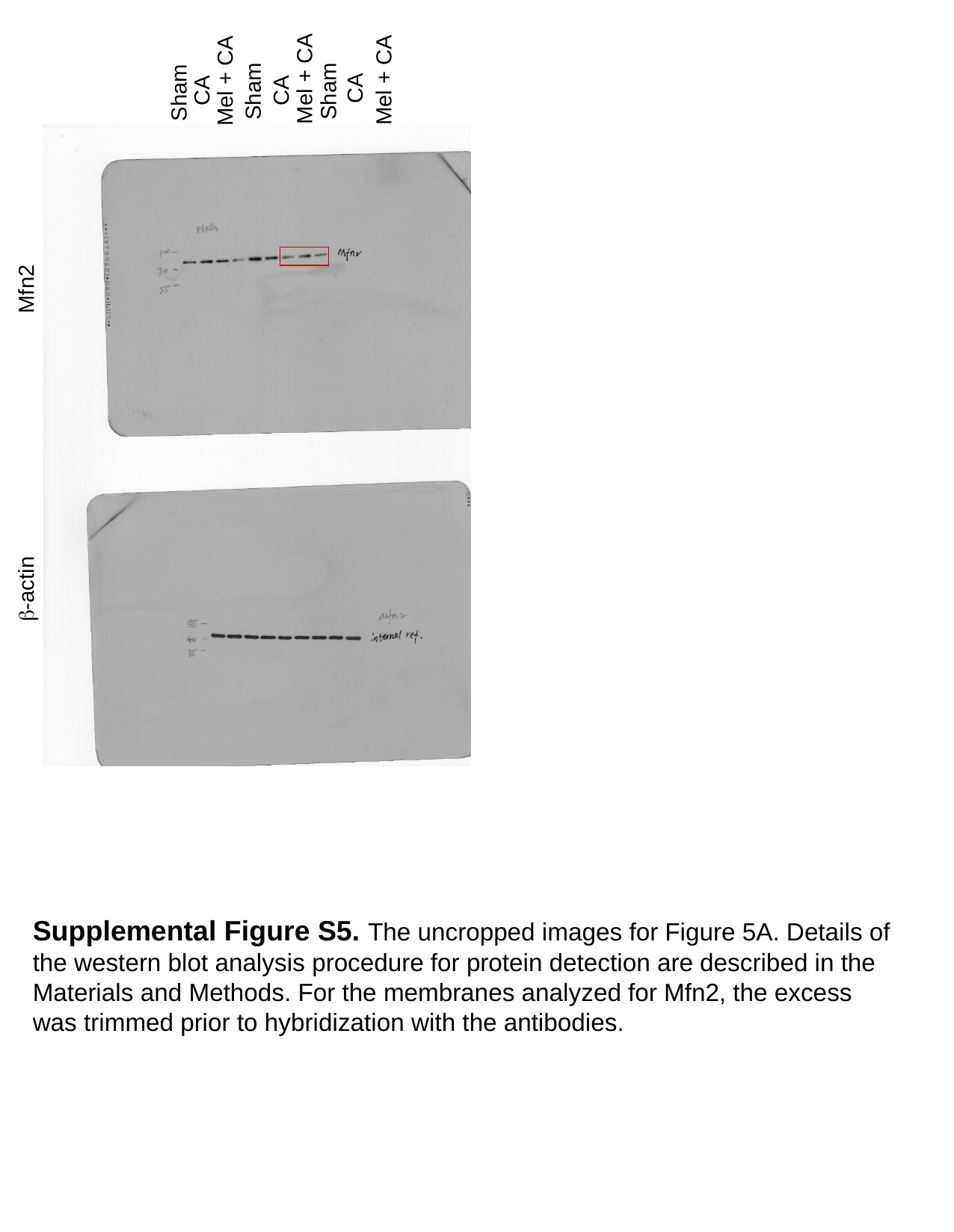

Mel + CA
CA
Sham
Mel + CA
Mel + CA
CA
CA
Sham
Sham
Mfn2
β-actin
Supplemental Figure S5. The uncropped images for Figure 5A. Details of the western blot analysis procedure for protein detection are described in the Materials and Methods. For the membranes analyzed for Mfn2, the excess was trimmed prior to hybridization with the antibodies.

## Slide 6
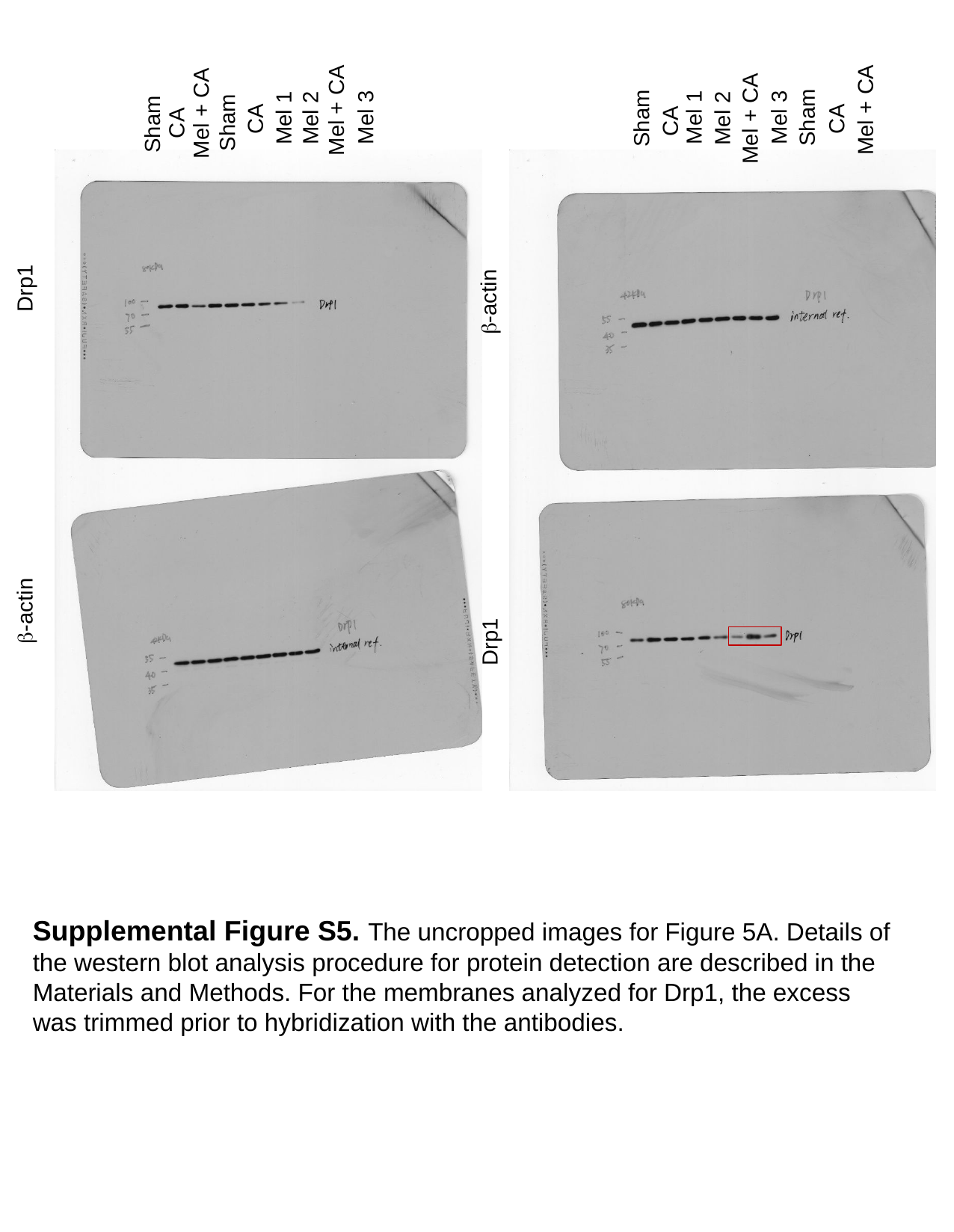

Mel + CA
Sham
Mel 3
Mel 1
Sham
Mel 2
Mel + CA
CA
CA
Mel + CA
Mel + CA
Mel 3
Mel 1
Mel 2
CA
Sham
CA
Sham
Drp1
β-actin
β-actin
Drp1
Supplemental Figure S5. The uncropped images for Figure 5A. Details of the western blot analysis procedure for protein detection are described in the Materials and Methods. For the membranes analyzed for Drp1, the excess was trimmed prior to hybridization with the antibodies.

## Slide 7
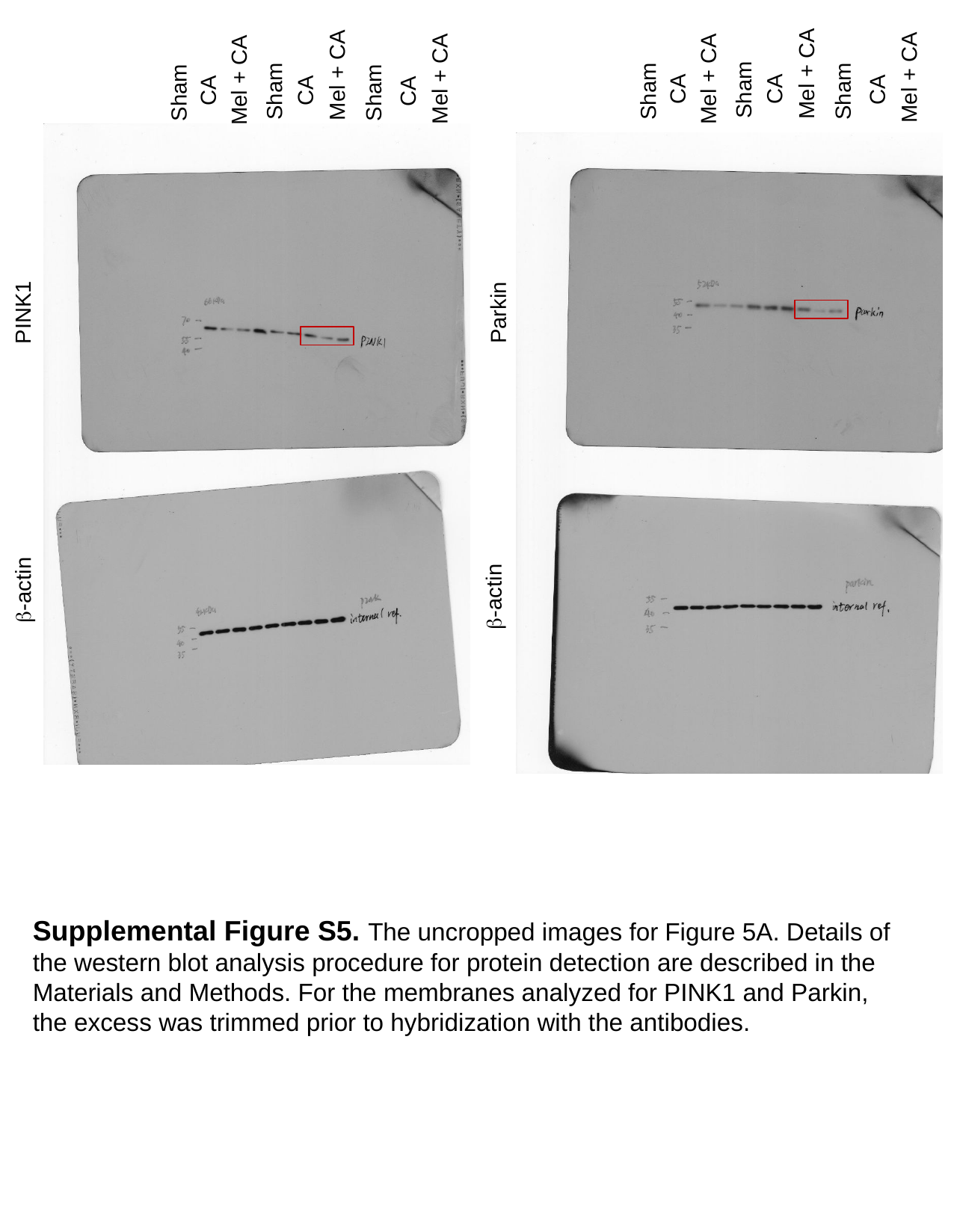

Mel + CA
CA
Sham
Mel + CA
Mel + CA
CA
CA
Sham
Sham
Mel + CA
CA
Sham
Mel + CA
Mel + CA
CA
CA
Sham
Sham
PINK1
β-actin
Parkin
β-actin
Supplemental Figure S5. The uncropped images for Figure 5A. Details of the western blot analysis procedure for protein detection are described in the Materials and Methods. For the membranes analyzed for PINK1 and Parkin, the excess was trimmed prior to hybridization with the antibodies.

## Slide 8
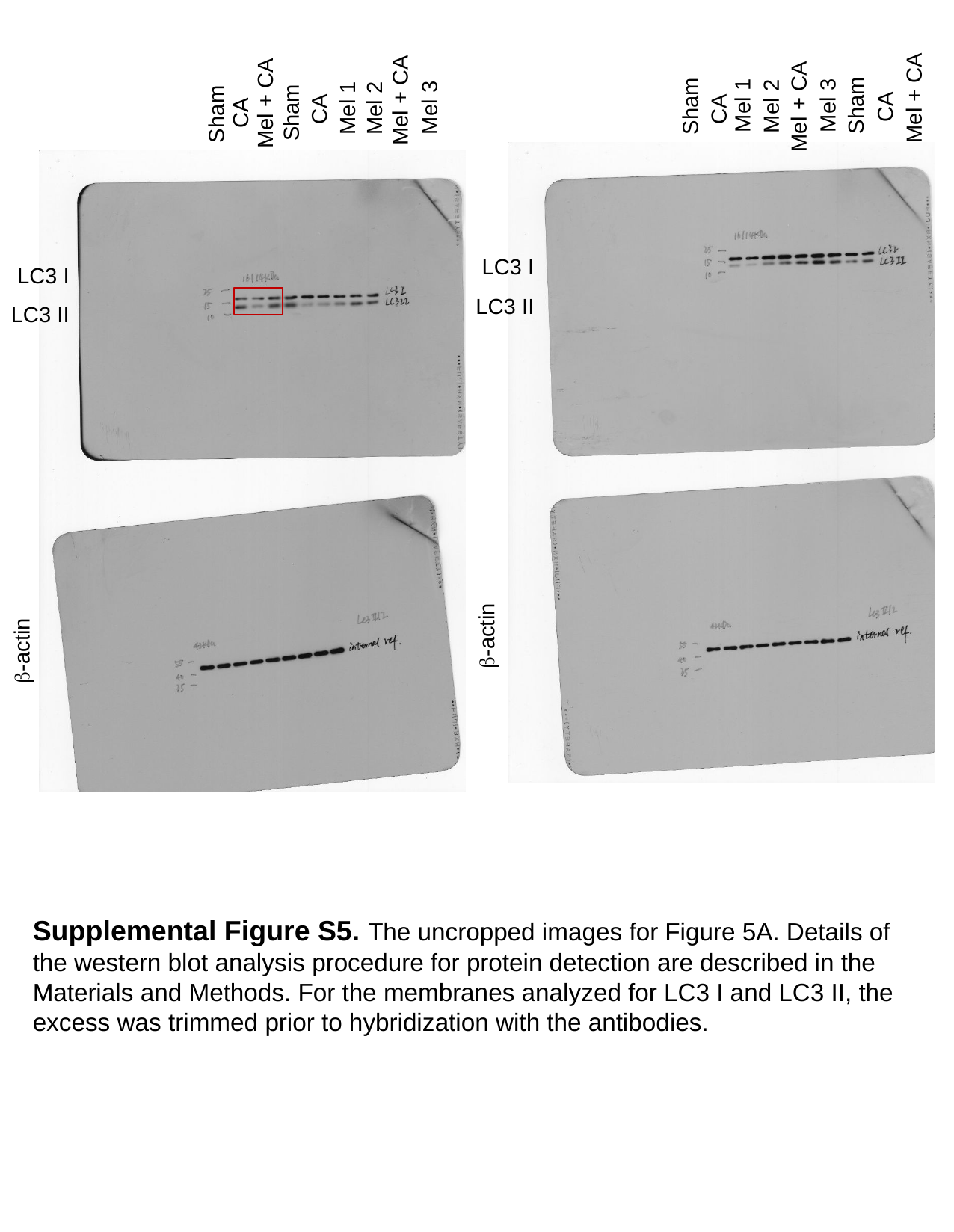

Mel + CA
Sham
Mel 3
Mel 1
Sham
Mel 2
Mel + CA
CA
CA
Mel + CA
Mel + CA
Mel 3
Mel 1
Mel 2
CA
Sham
CA
Sham
LC3 I
LC3 II
LC3 I
LC3 II
β-actin
β-actin
Supplemental Figure S5. The uncropped images for Figure 5A. Details of the western blot analysis procedure for protein detection are described in the Materials and Methods. For the membranes analyzed for LC3 I and LC3 II, the excess was trimmed prior to hybridization with the antibodies.
